# Surface feature based classification of plant organs from 3D laserscanned point clouds for plant phenotyping

**DOI:** 10.1186/1471-2105-14-238

**Published:** 2013-07-27

**Authors:** Stefan Paulus, Jan Dupuis, Anne-Katrin Mahlein, Heiner Kuhlmann

**Affiliations:** 1 Institute of Geodesy and Geoinformation - Professorship of Geodesy, University of Bonn, Nussallee 17, 53115 Bonn, Germany; 2 Institute for Crop Science and Resource Conservation (INRES) - Phytomedicine, University of Bonn, Nussallee 9, 53115 Bonn, Germany

**Keywords:** 3D-laserscanning, Surface feature histogram, Automatic classification, Plant phenotyping, High throughput, Wheat, Grapevine

## Abstract

**Background:**

Laserscanning recently has become a powerful and common method for plant parameterization and plant growth observation on nearly every scale range. However, 3D measurements with high accuracy, spatial resolution and speed result in a multitude of points that require processing and analysis. The primary objective of this research has been to establish a reliable and fast technique for high throughput phenotyping using differentiation, segmentation and classification of single plants by a fully automated system. In this report, we introduce a technique for automated classification of point clouds of plants and present the applicability for plant parameterization.

**Results:**

A surface feature histogram based approach from the field of robotics was adapted to close-up laserscans of plants. Local geometric point features describe class characteristics, which were used to distinguish among different plant organs. This approach has been proven and tested on several plant species. Grapevine stems and leaves were classified with an accuracy of up to 98%. The proposed method was successfully transferred to 3D-laserscans of wheat plants for yield estimation. Wheat ears were separated with an accuracy of 96% from other plant organs. Subsequently, the ear volume was calculated and correlated to the ear weight, the kernel weights and the number of kernels. Furthermore the impact of the data resolution was evaluated considering point to point distances between 0.3 and 4.0 *mm* with respect to the classification accuracy.

**Conclusion:**

We introduced an approach using surface feature histograms for automated plant organ parameterization. Highly reliable classification results of about 96% for the separation of grapevine and wheat organs have been obtained. This approach was found to be independent of the point to point distance and applicable to multiple plant species. Its reliability, flexibility and its high order of automation make this method well suited for the demands of high throughput phenotyping.

**Highlights:**

• Automatic classification of plant organs using geometrical surface information

• Transfer of analysis methods for low resolution point clouds to close-up laser measurements of plants

• Analysis of 3D-data requirements for automated plant organ classification

## Background

Aiming at high throughput plant phenotyping, one of the main challenges is the robust and automatic analysis of plant data [1]. In this context phenotyping implies the measurement of observable plant attributes, reflecting the biological function of gene variants as affected by the environment [2]. Whereby modern phenotyping techniques are used to study growth and development of large sets of plant genotypes under different stress situations [3,4]. In this connection 3D laserscanning allows a non-destructive assessment of various plant parameters under controlled conditions. The plant architecture, the plant height and size, specific plant organs or the organ volume can be deduced from the 3D structure of plants. A detailed evaluation of these parameters through time will help to link alteration in plant growth to stress tolerance, or to predict the yield potential of different genotypes.

Structural geometrical analysis of plants is an important technique to observe the development and growth of plants or the reaction of plants to abiotic and biotic stresses [5]. Highly resolved analysis enables the establishment of 3D organ based architectural models, like functional structural plant models [6,7]. The observation of subtle changes can be used to link geometrical deviation and deformation to environmental effects [8]. 3D-measuring devices like 3D-cameras, photogrammetric methods or laserscanners [6,8-10] provide non-contact and non-destructive 3D-measurements. However, only highly resolved and accurate 3D point clouds enable a valid description of the geometry of plant organs. Laserscanning results in huge point clouds with more than hundred thousand data points for a whole plant or ten to thirty-thousand points per plant organ [9]. This technique has been used in various studies for plant analysis [8,11] and stands out due to its quick, direct and automatic data collection [10]. Thus, the requirements for an implementation in phenotyping process are fulfilled.

The automatic recognition of shapes from point clouds is a prerequisite for plant phenotyping. The recognition of geometrical standard shapes like cylinders, spheres, planes or cones as well as combinations and variations is well described in various fields of research [12,13]. For 3D plant analysis the most common approaches use 3D mesh processing [8,14]. Paproki A, 2012 [14] used a 3D point cloud created out of 64 images of a cotton plant to detect single plant organs. Aiming at a segmentation of leaves, main stem and petioles, a region growing algorithm sensitive for curvature, noise, sharp edges and smoothness constraints was applied to a pre-calculated mesh. Furthermore primitive fitting algorithms were used to approximate organs like stems or petioles. The resulting regions were used for organ specific parameterization. This approach requires plant organs that can be abstracted by primitives and certain smoothness constraints e.g. of the leaf surface.

An entirely different approach that avoids mesh processing and uses more explicit properties to describe surfaces is the so called surface feature histogram [15,16]. Furthermore it overcomes the demands of smoothness constraints and the abstraction of primitives. These histograms enable a direct point classification by using descriptors for surface curvature and pointnormal-properties; moreover they provide an invariance to translation and 3D rotation. This technique has been optimized for the recognition of geometrical standard shapes [15], 3D point cloud registration [16], pose recognition [17] and for the recognition of kitchen objects like cupboards, tables and cups [18]. Points were linked to classes with similar surface properties by using Support Vector Machines (SVM) or Conditional Random Field (CRF) classification [19]. With this approach regions of similar points can be determined and a following model fitting can be applied to extract geometrical and functional maps of the environment [18]. This technique can be directly applied to a point cloud without calculating an additional surface representation as it is necessary for mesh-based approaches. However, it has to be demonstrated whether the recognition of point classes due to their surface properties and the extraction of geometrical maps can be transferred to various situations in plant research. This task is of high importance for plant phenotyping, where high throughput laserscans of plants can be used to extract growth curves of specific plant organs [3].

Surface feature histograms have shown their applicability for online procession in robotics. The transfer of this method to plant phenotyping promises huge benefits to speed up phenotyping processes with high accuracy. Especially the application to highly resolved close-up laserscans from plants has never been realized before. Until now a descriptive representation of the local geometry of plant point clouds by surface feature histograms has not been applied to complex structures like plant organs (e.g. leaves stems or wheat ears). The establishment of 3D measuring devices for plant imaging during the past years and upcoming high precision laserscanning methods particularly evoke the demand of specified and adapted techniques and algorithms for point cloud processing [8,14]. Certainly laserscanning provides Euclidian XYZ-data with device specific differences in the point to point distance (hereafter resolution), the amount of occlusion and in the accuracy. Hence a fast and accurate data processing method is required for the implementation in an automatic measuring and classification workflow. Previous experiments show the demand for algorithms aiming at an automated classification [7,14] without specific requirements on the shape [20] or on additional sensor data [21].

In the present paper, we introduce an adapted surface feature based technique for an automated pointwise classification of plant organs from 3D laserscans. We show an automated separation of grapevine point clouds to the plant organs leaf and stem, as well as a separation of wheat ears and stems to extract yield parameters. Furthermore, the impact of the point cloud resolution was evaluated with respect to the classification accuracy.

## Results

The main focus of this study is the processing of 3D point clouds, which do not provide any additional information for the classification like e.g. color. We aim at a geometry based labeling of single points, which can be used to define regions of similar geometry, describing underlying plant organs as it is important for parameterization. The three key outcomes of this methological study are i) the adaption of a low resolution algorithm on the demands of highly resolved point clouds for grapevine plant organ classification, ii) an empirical evaluation of different point resolutions to show the validity for different kinds of 3D-measuring devices and iii) the integration of the proposed method in a processing workflow for an automated yield calculation of wheat plants.

### Plant organ classification by feature based histograms

**Figure 1:**
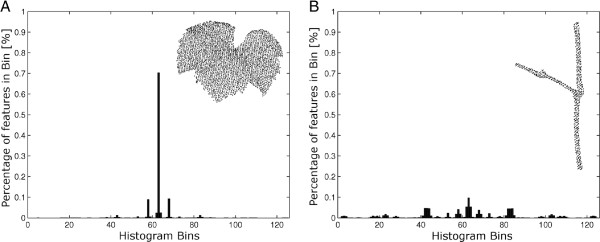
**Histogram for the the laserscanned point cloud of a grapevine leaf (A) and of a grapevine stem point cloud (B).** Histogram calculation was applied using *r*_*N*_=2.5 *mm* and *r*_*H*_=3.5 *mm* and a point cloud with a resolution of 0.3 *mm*.

Surface feature histograms show unique characteristics for point clouds that differ in the euclidean properties of their surface. Figure 1 introduces the geometrical descriptions of the surface properties of two point clouds of a grapevine leaf (A) and a grapevine stem (B), visualized as surface feature histogram. The algorithm of [16] calculates surface feature histograms using pointwise neighbor features. To increase the descriptive properties of surface feature histograms even with large histogram radii, we introduced a distance weight for the calculation. Subsequently, these histograms were used as features for SVM classification.

The classification results of a grapevine scan for different point cloud resolutions, from 0.33 *mm* to 4.0 *mm*, is shown in Table 1. The mean results of a repeated random sub-sample cross-validation using ten iterations are presented. 4% of the points were randomly chosen training data for each class. This approach provides a reasonable prediction as it is necessary for real applications. A resolution of e.g. 1.0 *mm* implies that the point cloud shows minimal point to point distances of not less than 1.0 *mm*. The normal for every point has to be calculated due to the output of laserscanners pure 3D point clouds. Therefore the neighbors of a source point within the radius *r*_*Normal*_ = *r*_*N*_ were used. Comparable to this, the calculation of the surface feature histogram only considered points within radius *r*_*Histogram*_ = *r*_*H*_. Each column of Table 1 describes the results for the combination of different *r*_*N*_ and *r*_*H*_ varying from $\frac{1}{3}\;\text{mm}$ to 5 *mm* in steps of $\frac{1}{3}\;\text{mm}$ for both variables. Best performing combinations of *r*_*N*_ and *r*_*H*_, for each resolution, were evaluated. As initial values for *r*_*N*_, the value slightly bigger than the point cloud resolution was used and as maximum value the radius of the smallest object - here the stem diameter (5.0 *mm*) was chosen. The histogram algorithm requires a *r*_*N*_ that is smaller than *r*_*H*_, but bigger than the point cloud resolution. Due to this restriction the amount of combinations decreases with increasing point resolution. Thus resolutions above 4.0 *mm* result only in one specific value or were not calculable. The best classification results were shown together with related *r*_*N*_ and *r*_*H*_ values. The classification results were validated using manually distinguished data, labeled by Geomagic Studio 12 64Bit (Raindrop Geomagic Inc, Morrisville, NC, USA).

**Table 1:** **Impact of point cloud resolutions (0.33 *mm *to 4.0 *mm*) to the classification result and the calculation time**

**Point cloud resolution [mm]**	**0.33**	**0.66**	**1.0**	**1.33**	**1.66**	**2.0**	**2.33**	**2.66**	**3.0**	**3.33**	**3.66**	**4.0**
classification result (SVM) [%]	97.9	98.3	98.1	98.0	97.8	97.2	96.6	96.1	95.8	95.3	94.7	93.2
best *r*_*N*_ [mm]	1.33	1.6	1.6	2.0	2.3	3.0	4.0	4.0	4.3	4.3	4.6	4.6
best *r*_*H*_ [mm]	5.0	5.0	5.0	5.0	5.0	5.0	5.0	5.0	5.0	5.0	5.0	5.0
misclassified points (in thsnd.)	10.6	2.3	1.05	0.7	0.5	0.4	0.4	0.4	0.3	0.3	0.3	0.3
points (in thsnd.)	527.0	134.3	55.6	35.4	24.4	16.3	12.5	10.0	7.7	6.4	5.4	4.4
points of leaves (in thsnd.)	407.8	108.0	45.3	29.1	20.1	13.5	10.5	8.4	6.5	5.5	4.6	3.8
points of stem (in thsnd.)	119.2	26.3	10.3	6.3	4.3	2.8	2.0	1.6	1.2	0.9	0.8	0.6
mean calculation time [sec]	1160	155	52.2	31.1	19.8	12.3	9.5	7.4	5.6	4.7	4.07	3.3
min calculation time [sec]	465	105	40.6	26.1	17.9	11.7	9.1	7.2	5.6	4.7	4.0	3.2
max calculation time [sec]	1788	184	58.3	34.6	22.5	12.8	9.7	7.4	5.7	4.7	4.1	3.4

Satisfying classification accuracies of ≥90*%* were achieved for point cloud resolutions between 0.33 *mm* and 4.0 *mm*. The best classification results above 96% were constantly reached for resolutions smaller than 2.66 *mm*. Indeed the best classification accuracy of 98.3*%* was found at a point cloud resolution of 0.66 *mm* using *r*_*N*_ = 1.6 *mm* and *r*_*H*_ = 5.0 *mm*. Furthermore, the classification results for different point cloud resolutions depends on the combination of *r*_*N*_ and *r*_*H*_. For each point cloud resolution the best classification results can be reached by using a large radius *r*_*H*_. While this tendency is valid throughout all different resolutions, this can not be generalized for *r*_*N*_.

Furthermore, we perceive a slow decrease in the classification accuracy with a decreasing resolution. This is strongly connected with a decreasing number of points providing the point cloud. At a resolution of e.g. 0.33 *mm* 527092 points were considered, 12500 for a resolution of 2.33 *mm* and 4000 points at the lowest resolution of 4.0 *mm*. Considering the ground truth data, approximately 80% of all points belong to the grapevine leaves and 20% to grapevine stems for throughout the used point cloud resolutions. At the highest resolution of 0.33 *mm* more than 10 thousand points were mislabeled. This amounts to only 2.1*%* of the respective 527092 considered points. A similar percentage of misclassified points were obtained at higher resolutions.

**Figure 2:**
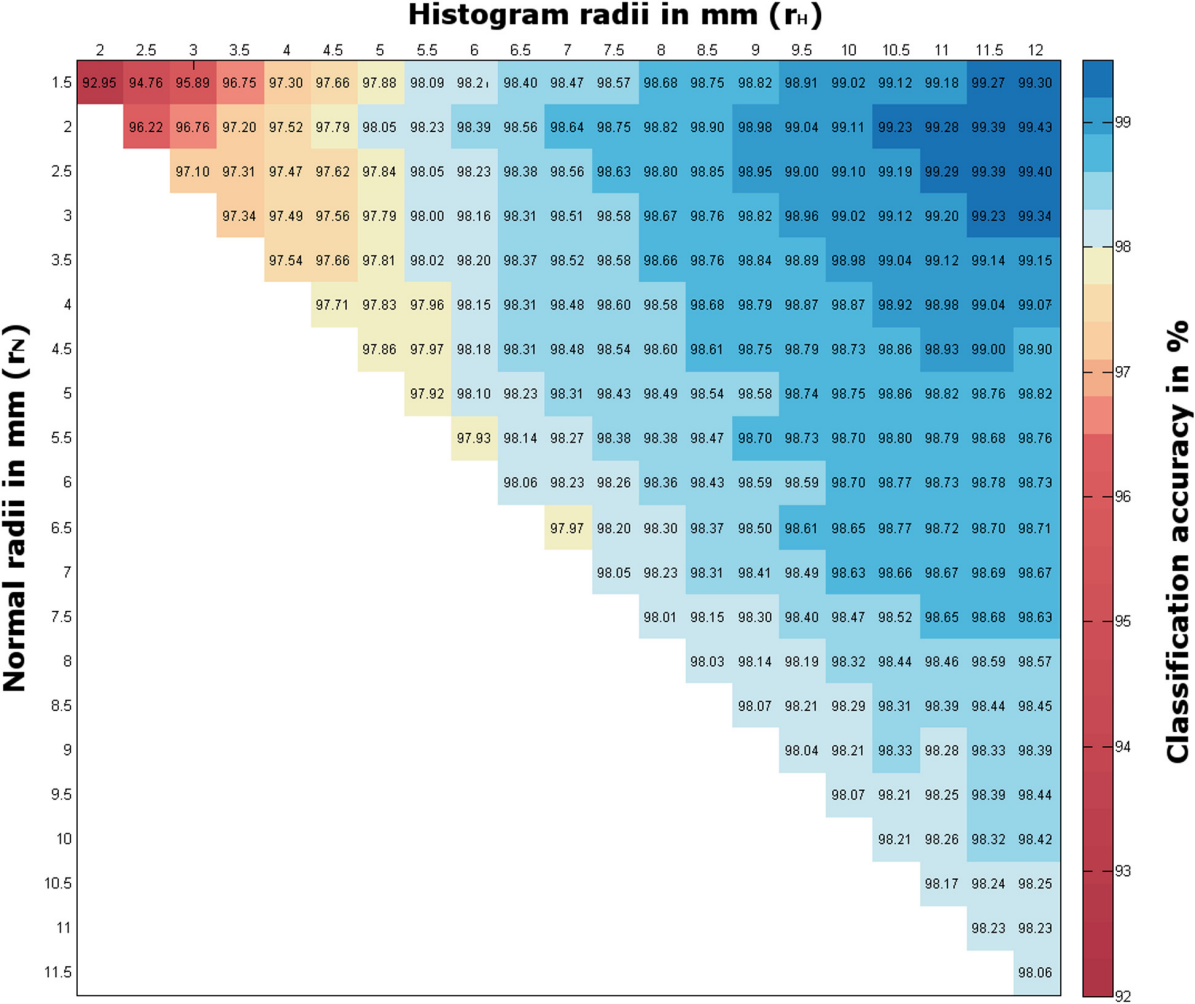
**Heatmap for the classification results according to different histogram and normal-radii of grapevine point data with a resolution of 1 *mm*.**

Further analysis showed an influence of the parameters *r*_*N*_ and *r*_*H*_ to the classification accuracy. The variation in classification accuracy for *r*_*N*_,*r*_*H*_ ≤ 12 *mm* and a fix point cloud resolution of 1.0 *mm* is shown in Figure 2. A resolution of 1.0 *mm* was chosen exemplary for the visualization in Figure 2, due to its satisfying results regarding calculation time and classification accuracy. Our aim is a deeper understanding of the impact of the used radii *r*_*N*_ and *r*_*H*_ to the classification result. The best classification accuracy of more than 99% can be found within a small radius of *r*_*N*_ between 1.5 *mm* and 3.0 *mm* and a *r*_*H*_ of 9.5 *mm* to 12 *mm*.

**Figure 3:**
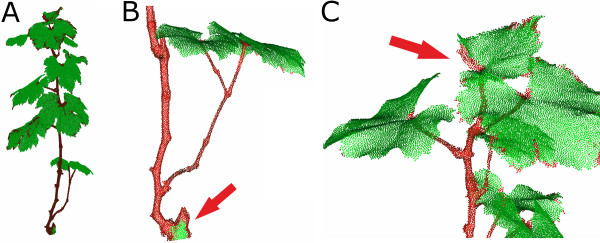
**Classification of grapevine leaves (green) and stems (red) (A).** Misclassification appears in regions of plain stem surfaces **(B)** as well as at leaf borders **(C)**, shown by red arrows. The classification was done using a normal radius of 2.5 *mm* and a histogram radius of 12 *mm* with a point cloud with 1 *mm* point resolution.

In Figure 3 (A) a detailed view on a classified grapevine point cloud with a point resolution of 1 *mm* is shown. Using *r*_*N*_ = 2.5 *mm* and *r*_*H*_ = 12 *mm* an accuracy of about 99% has been reached. Unfortunately, this means that 417 of 55635 points have a wrong classification label (Table 1). Figure 3 (B) and (C) show typical misclassification of the plant organ. Points that belong to a grapevine stem (red) are misclassified in regions where we locate a surface geometry very similar to a leaf surface (green) geometry (Figure 3B). *Vice versa* parts of the leaf surface are classified as stem, especially at the transition between leaf and stem and in the border area of the leaf (C).

We have introduced surface feature histograms together with SVM classification as a method for a highly accurate separation of plant organs of a grapevine plant. We determined *r*<*r*_*N*_<<*r*_*H*_ with *r* represents the point clouds resolution. Using histogram radii of 9.5−12 *mm* leads to a satisfying covering of the points neighborhood. This results in a high classification accuracy of about 99%. The resolution should be choosen with respec to the expansion of the smallest object which has to be classified. Thus, the resolution of the grapevine point cloud shouldn’t be bigger than the minimal diameter of the stems.

### Wheat yield estimation by online processing

Previous results have shown histogram based classification for 3D point clouds of grapevine for an automated extraction of plant organs such as leaf and stem. In the following subsection we transfer previous findings to the classification of 3D point clouds of wheat plants, to determine stem and ear points automatically. This method was integrated in a workflow for an automated volume calculation of wheat ears, which is of importance for wheat yield estimation and prediction. It shows the applicability of 3D laserscanning in high throughput phenotyping.

**Figure 4:**
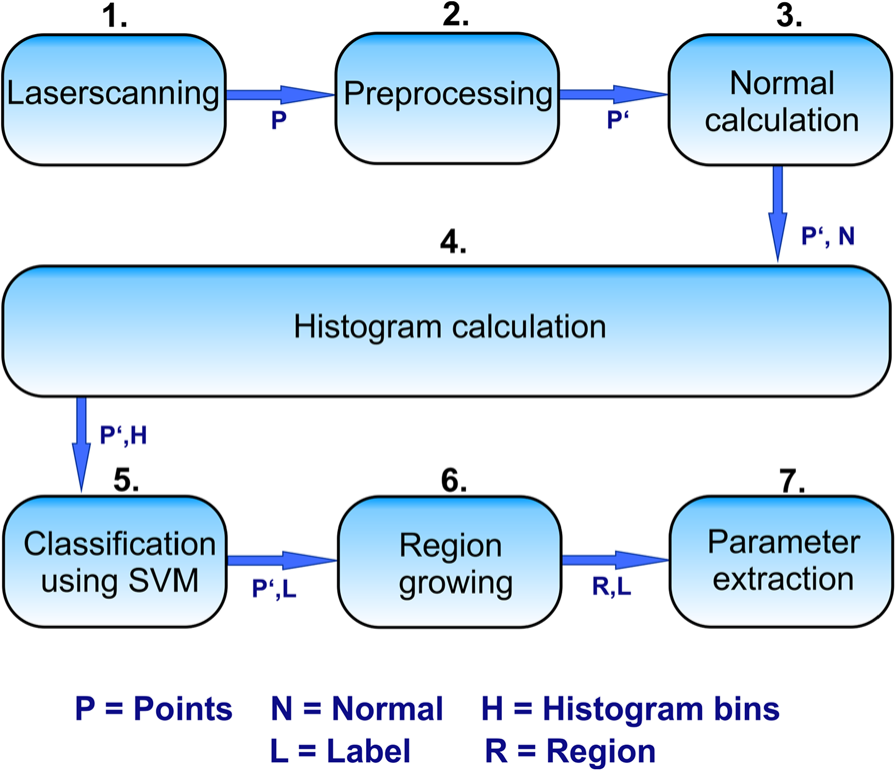
**Dataflow diagram showing the single steps for automated online measurements of wheat yield parameters 1) Laserscanning, 2) Preprocessing, 3) Normal calculation 4) Histogram calculation, 5) Data classification, 6) Region growing and the final 7) Parameter extraction.** Arrows indicate the data, while boxes describe the processing steps.

**Figure 5:**
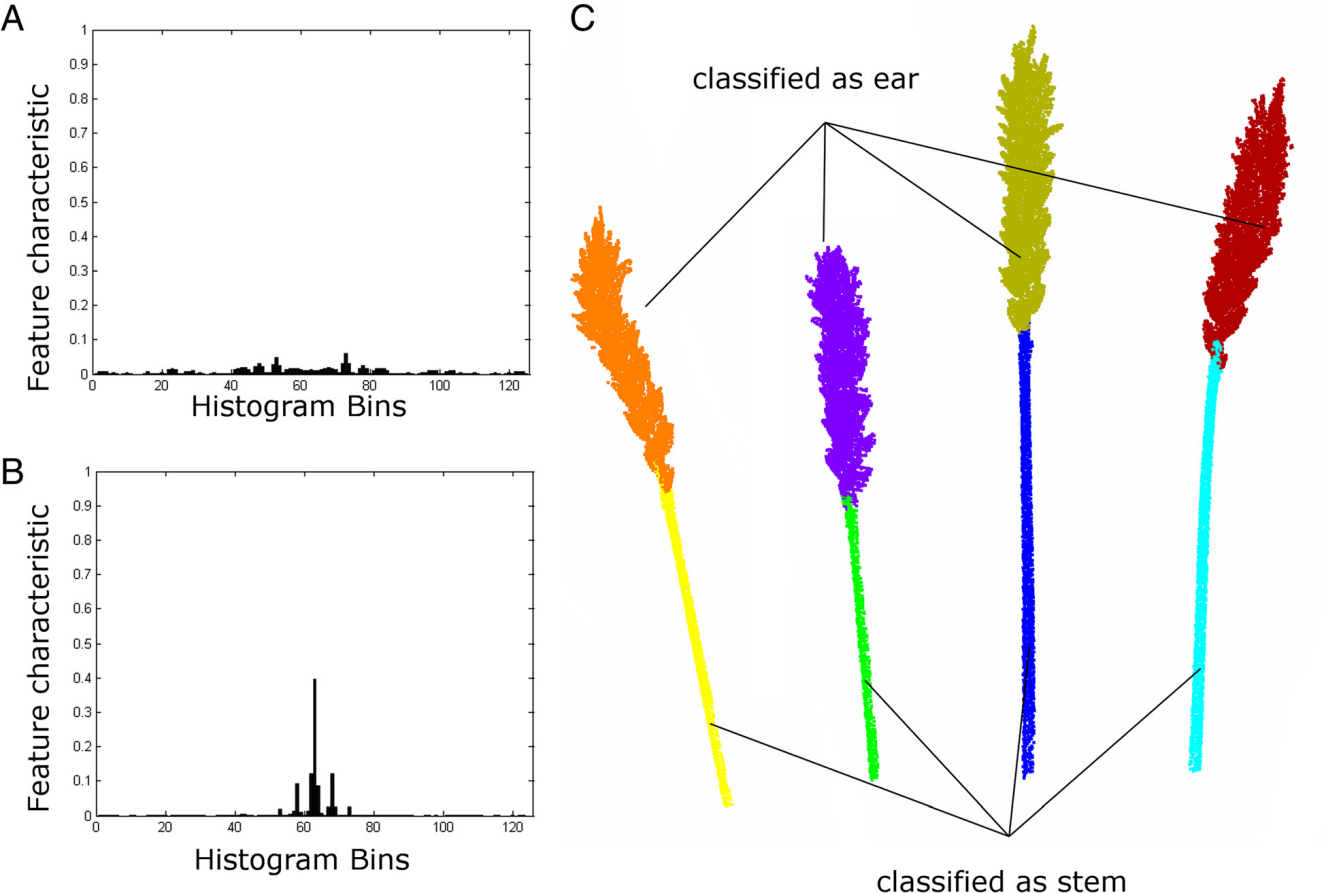
**Histograms of wheat ear (A) and stem (B) and the classified point cloud of a wheat plant using *r***_***N ***_**= 2.5 *mm*, *r***_***H ***_**= 12 *mm*.** A region growing algorithm was applied for automatic elimination of wrong classified small regions **(C)**. Colors indicate regions which are belonging together.

A wheat point cloud with a resolution of 1.0 *mm* was used for further processing, in accordance to our experience from grapevine plant organ classification. For normal- and histogram calculation *r*_*N*_ = 2.5 *mm* and *r*_*H*_ = 12 *mm* were used. The processing pipeline is as follows 1) laserscanning, 2) pre-processing including cutting of pot points and leaf points, 3) normal calculation, 4) histogram calculation, 5) classification using SVM, 6) region growing and 7) parameter extraction. A visualization of the dataflow is shown in Figure 4. Steps 1 to 5 have been outlined before and were described in the subsection above, therefore we focus on the last two steps to detect the different regions of the labeled point cloud. It was assumed that regions of interest have a significantly bigger size than mislabeled regions. Thus, smaller regions are mislabeled and can be connected to bigger regions next to them. This was done using a region growing algorithm. The results can be seen in Figure 5. The left side shows a characteristic histogram for wheat ears (A) and wheat stems (B) that were calculated out of the training data and used for subsequent SVM classification. Figure 5 (C) shows the results of the classification process of one plant after the region growing step. Separated by colors, the regions are visible. Originally the classification resulted in 39 regions. These were reduced to 8 regions by region growing, clearly dividing 4 ear and 4 stem regions. Overall we reached a mean classification accuracy of 96.56*%* at a calculation time of 5.40 minutes and 65 thousand points to classify eleven of twelve wheat ears using a leave-one-out cross-classification method. A clear separation of a wheat laserscan was shown using surface feature histograms. The points were allocated to the classes ear and stem and aggregated to a relevant region size. This was done fully automated and enabled the application of a volume estimation algorithms.

**Figure 6:**
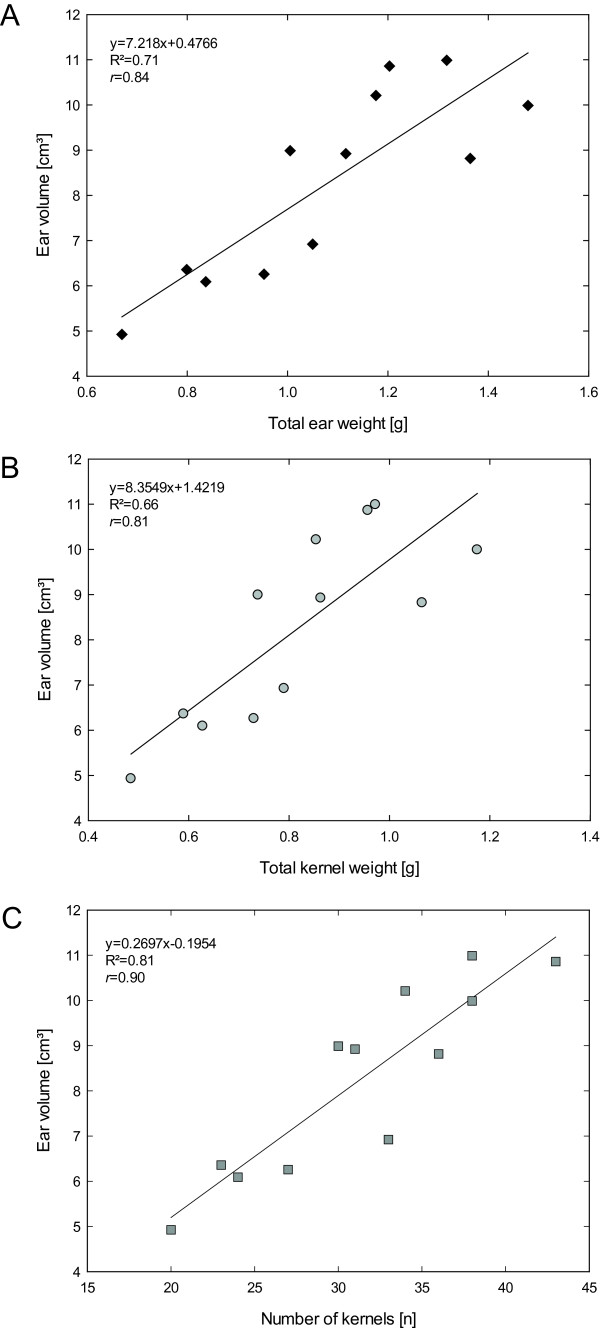
Correlation of the volume of the single ears in relation to the total ear weight (A), the total kernel weight (B) and the number of kernels (C).

After the classification of different plant organs quantitative plant parameters were deduced from 3D laserscans. An alpha shape volume estimation was applied to the organ regions. This method enabled an easy and fast way for volume estimation and an accurate description of the concave wheat ears. These parameters were related to manually assessed yield parameters. Significant correlations were found between the measured ear volume and *de facto* yield parameters. The parameters total ear weight, total kernel weight and number of kernels showed high correlations of R^2^ = 0.71, R^2^ = 0.66, and R^2^=0.81, respectively (see Figure 6).

## Discussion

The main goal of the current research was to find a fast and accurate technique for the classification of different plant organs out of laserscanned plant point clouds. A method from robotics [15,16] was modified and extended to the demands of plant organ classification from high precision laserscans for plant phenotyping. It was applied to the classification of grapevine point clouds to determine leaf and stem points,and to separate wheat point clouds into stem and ear points. Both point cloud classification problems were solved with a high accuracy of more than 96*%* within a relatively short calculation time of a few minutes.

Separation of plant organs was reached by a new surface description method. Our method trains the local geometry of the organs and can e.g. be used for classification of various plants by using only one single manually labeled plant. Previous research used a pre-calculated mesh [3,6] with special surface assumptions like smoothness constraints or approximation by primitives to separate single plant organs. We were able to reduce the amount of external knowledge required for classification and to avoid mesh calculation by adapting a method based on the points itself. However, difficulties arise when the transition is not clearly defined between different plant organs. This is the case between a leaf and a stem or for plain stem regions which are similar to a plain leaf surface. Here the results are small regions with a wrong label. To overcome this effect, we implemented a region growing algorithm following the assumption that smaller regions received an incorrect classification label. This was successfully implemented for the classification of large connected regions as shown for wheat ears and grapevine leaves, but fails for the classification of smaller regions like e.g. leaf veins. The classification workflow results in regions describing the single plant organs that can be used for a direct parameter extraction, such as e.g. ear volume. An accurate and early estimation of grain yield is desirable for plant breeding or agrobusiness. In plant breeding, genotypes with high potential yield have to be selected in high throughput. Yield estimation in the field is required e.g. for planning harvest and storing requirements, for cash flow budgeting or for crop insurance purposes. Until now extensive personal experience is essential for visually estimating yield of cereal crops, alternately destructive assessment is the method of choice [22]. Our laserscanning approach can substitute traditional yield estimation techniques. Principal benefits are the objectiveness, the high accuracy and reproducibility. Separation of single organs is the key to enable plant parameterization on the organ level.

The proposed method can be applied to different plant types and different organ geometries. Previous research with stereo camera systems [6,23] or Time of Flight cameras [24,25] is supported as well as laserscanning devices with various point resolutions [8,10]. This has been proven by the reduction of the point cloud resolution, still resulting in satisfying results (Table 1). Seitz S, 2006 [26] showed that algorithms for multi-view stereo reconstruction improve rapidly and provide a point accuracy that is only slightly lower than the accuracy provided by the laserscanning. Furthermore [15,27] had shown its applicability for the use of noisy point clouds with a very low accuracy. Hence, the method is independent of the used 3D imaging sensor.

Aiming at an integration in existing high throughput phenotyping environments a deeper look into the calculation time is necessary. The computational effort is closely linked to the number of points (n) and the number of points in the neighborhood (k). The bigger the used histogram radius and the higher the point cloud resolution, the more points influence the histogram calculation. This leads to a computational complexity of *O*(*n*∗*k*) as it was shown by [16]. We can confirm this assumption by our results. E.g. reducing the resolution from 0.33 *mm* to 0.66 *mm* results in 25*%* of the original number (n) of points and calculate histograms with the same radius of e.g. 5.0 *mm*, the calculation time is reduced to about 10*%* compared to 25*%* of the original time. This can be explained by a decrease in the number of points that have to be considered in every calculation step (*k*_0.33_≤*k*_0.66_). The calculation time for the histogram based approach is comparable to the processing time and thus it is well suited for online processing. Compared to [28] who used 2-D images to assess the leaf area of different arabidopsis genotypes, our approach enables an automated labeling of wheat and grapevine plants in less than two minutes for a resolution of 1 *mm* in 3D.

With respect to a fast and optimized calculation time - which can still be improved by e.g. faster implementation using the computers graphical unit - the method is well suited for the demands of automated high throughput phenotyping. These platforms collect an increasing amount of data, temporarily and spatially highly resolved [29]. An automated data processing method for high resolution point clouds is needed for classifying and characterizing various plant organs. Beyond this scope our method can be seen as a generalized approach for high throughput plant parameterization. Current methods [23,24] can be improved by adding surface properties to the organ separation step without calculating a triangle mesh or special requirements regarding e.g. smoothness.

Our method enables an automated classification of plant organs for plant parameterization. This can be implemented as an autonomous work package in a phenotyping process. Based on the presented approach, a database with class-specific training data can be introduced, where expressive histograms are used for the classification of unknown point clouds. This will improve the modeling of plants [7,30] which in turn can be used to improve the classification due to knowledge of the structure rules of a plant and its organs. The proposed method provides outstanding potential to be implemented in a sensor fusion approach for plant phenotyping or screening processes with optical devices [4,31]. Future research will concentrate on linking 3D-laserscans with imaging sensor data such as hyperspectral imaging or thermography to improve the accuracy in observing the impact of abiotic or biotic factors on plant physiology and on the plant phenotype.

## Conclusions

Automated organ parameterization is of high importance for plant phenotyping. We demonstrated that this can be realized using 3D point clouds without applying any mesh processing algorithm. Only little apriori knowledge of the plant organ surface is required, which can be trained independent of the data. We obtained highly accurate results for organ classification of a grapevine plant by using surface feature histograms. Furthermore, our approach was applied to wheat ear parameterization, which was compared to manually measured yield parameters.

The strength of our approach is the flexibility for an application to various 3D measuring devices and it can be generalized for the classification of different plants and plant organs. Automated and reproducible characterization of various plant 3D point clouds with high accuracy and its integration in high throughput phenotyping procedures was realized. Future research will concentrate on enhancing the geometrical sensitivity. Furthermore we will improve the direct parameterization of various organs like stem, ears and leaves at the same time and in one processing step by using multi-class classification.

## Methods

### Data Acquisition

**Figure 7:**
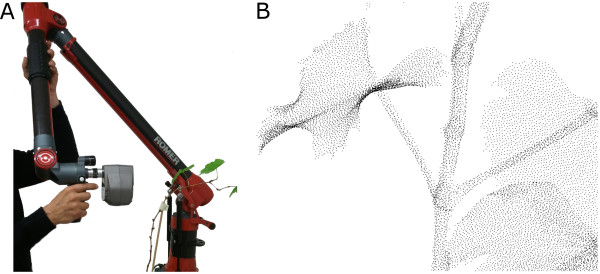
Laserscanner-measuring arm combination (A) and 3D data of grapevine (B).

3D point clouds were acquired by a high resolution laserscanner (Perceptron Scan Works V5, Perceptron Inc., Plymouth MI, USA) using an active laser triangulation method. The system was mounted on an articulated measuring arm (Romer Infinite 2.0 (2.8*m* Version), Hexagon Metrology Services Ltd., London UK) to enable an automated fusion of single scan-lines, coming from different points of view (Figure 7A). Thus, point clouds could be acquired with a minimum of occlusion (Figure 7B). The 3D laserscanner has a measuring field of 110 *mm*×105 *mm*, providing a point reproducibility of 0.088 *mm*. It was chosen due to its high point resolution, leading to highly reliable point measurements. The high resolution and accuracy is fundamental for organ specific classification and precise measurement of plant deformation as it is focused in phenotyping.

To prepare the raw data for classification, we used the commercial software Geomagic Studio 12 (Raindrop Geomagic Inc, Morrisville, NC, USA). This preprocessing included: i) cutting off parts that were scanned but do not belong to the plant, ii) rasterization of the point cloud to avoid a heterogeneous point distribution due to the scanners manual affected motion during scanning, and iii) the manual assigning of ground truth data. For the processing of the grapevine we used the complete point cloud beginning at the top of the pot and for the analysis of the wheat we focussed on the points above the highest leaf. This reduced the complexity of the classification by only using wheat ears and stems instead of ear, stem and leaves.

### Surface feature histograms

**Figure 8:**
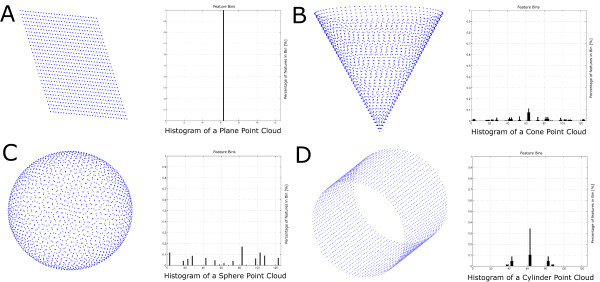
Point clouds of geometrical standard shapes like plane (A), cone (B), sphere (C) and cylinder (D) show unique histograms due to their differing surface properties.

3D laserscanned point clouds were analysed by surface feature histograms. They were developed by [15,16] for the demands of robotics and adjusted for classification of geometrical primitives in low resolved point clouds. The surface feature histograms are well suited for real time processing of laser data. Furthermore they provide a density and pose invariant description of the surface using properties of differential geometry. They can be used for point cloud segmentation and separation of different surface areas showing different surface properties. Histograms descriptions for primitives like plane, cube, sphere, cylinder and cone are shown in Figure 8. The characteristic of a histogram to a single point depends, beside the properties of the surface, on the radius for normal calculation *r*_*N*_ and the radius for the histogram calculation *r*_*H*_. Both parameters represent the number of neighbor points that were used for calculation. The histogram implementation follows an adapted approach using a special distance weight explained below.

**Figure 9:**
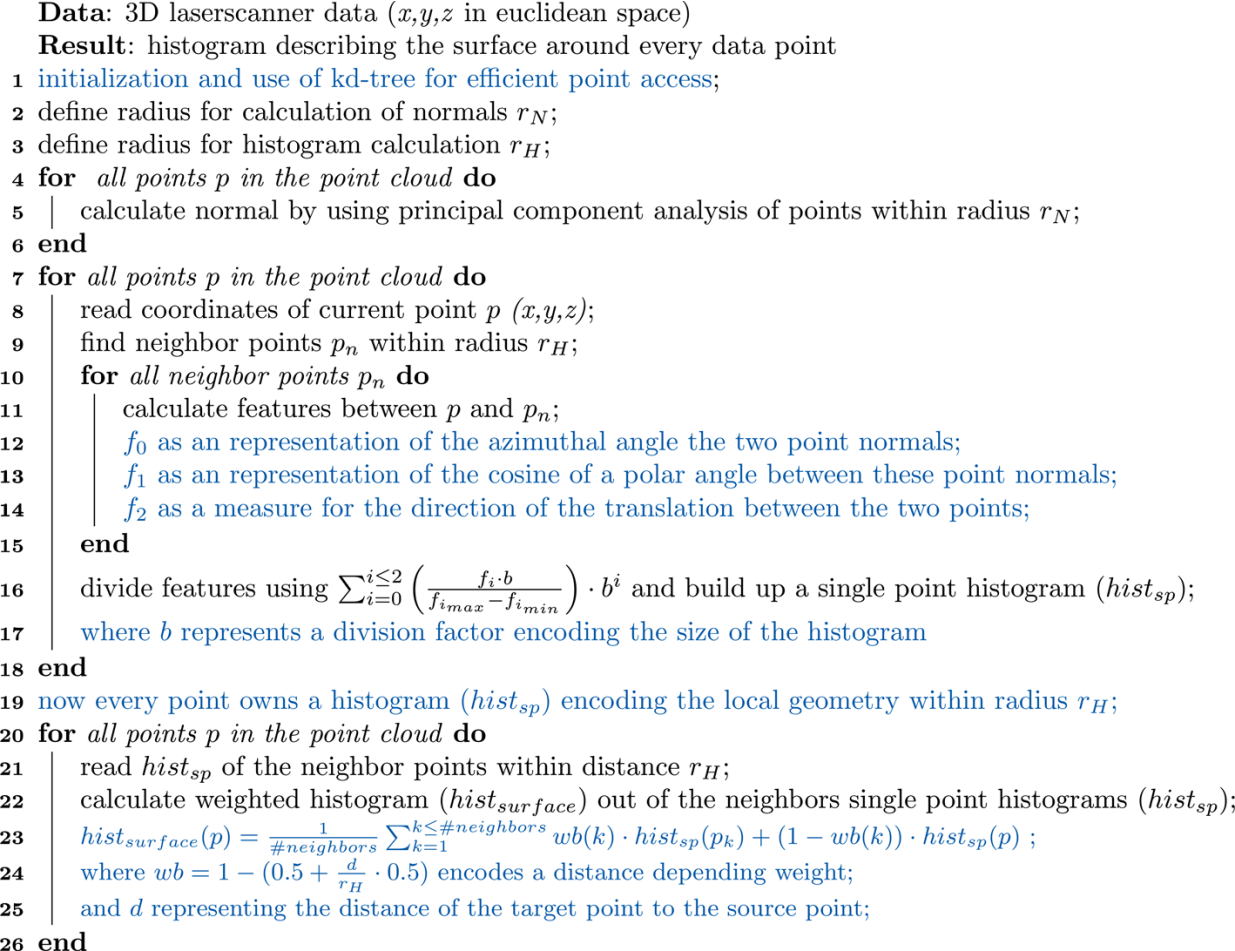
**Pseudocode for the calculation of surface feature histograms for pointwise surface description, modified from **[16]**.** Black letters denote commands, comments are indicated by blue letters.

As described by [16,19] and to find in Figure 9 line 20, a histogram to a source point consists of the weighted sum of the histograms to the neighbor points. The use of the original weight results in non-normalized histograms which complicate the use of support vector machines (SVM) for classification. Therefore a more detailed weight is needed for the classification of complex structures like stems, leaves, or ears instead of geometrically simple shapes like primitives.

(1) $$\begin{array}{lcrr} \text{wb}=1-(0.5+\frac{d}{r_{H}}\cdot 0.5) & & & \end{array}$$

We introduced a distance depending weight, see Equation 1, to take these disadvantages into account. The parameter *d* denotes the distance between the target point and the source point. Histograms of points, with a distance near the limit of the radius for histogram calculation (*r*_*H*_), have a very low impact for the final feature histogram calculation. This impact is raised as the distance to the source point becomes smaller. For points with a distance *d*→0 the weight raises up to an equal weight as the source point (50:50). For online processing of wheat plants for yield estimation a dataflow was designed and successfully adapted; the single processing steps are shown in Figure 4. It shows the steps 1) laserscanning of the plants, 2) preprocessing of the point cloud, 3) calculation of the point normals, 4) calculation of the histogram 5) classification of stems and ears using SVM with histograms of the training data 6) using a region growing algorithm to extract regions and to connect smaller regions to the next bigger ones and 7) calculation of the alpha shape volume as a measure for the parameter for yield estimation.

Since its introduction by [32] SVM were used for various aspects of classification in agricultural context [33]. It turned out to be a powerful machine learning technique that can be used for general-purpose supervised prediction [34]. Reliable results have already been achieved with the combination of surface feature histograms and this classification technique. About 4% of the points of every class were used as training data for grapevine classification. The amount of points was equivalent to the number of points of one complete leaf and a significant part of the stem in average. For the study of the resolution behaviour of the histograms a repeated random sub-sampling validation was calculated using ten iterations. For the classification of the twelve wheat plants a leave-one-out cross validation was used. One stalk was labeled and used for classification of the eleven remaining stalks. The mean results for twelve repetitions using each stalk for training were presented. While the results show the impact of a varying *r*_*N*_and *r*_*H*_for the classification of a grapevine point cloud, the classification of the wheat plants followed the parameters gained in this first part. A normal radius *r*_*N*_of 2.5 *mm*and a histogram radius *r*_*H*_of 12 *mm*were used for classification. They were chosen due to accuracy and calculation time (Table 1).

The postprocessing after classifying the 3D point clouds (Figure 4, step 5) included a region growing algorithm (Figure 4, step 6) that merged the points of a smaller region to a bigger region in the direct neighborhoods. We used the regions next to the centroid of the smaller region. Small regions were defined as regions that have less than the mean of the region sizes resulting from the region growing step after classification (see the explaining movie in the Additional file 1).

Calculating the alpha shapes of the ear regions (Figure 4, step 7) enabled a volume parameterization of the ears. An implementation, which is available for free at the Matlab exchange software website by Jonas Lundgren (2010) was used. It enabled a much more precise volume estimation than e.g. the convex hull and led to the extraction of wheat yield parameters almost automatically out of a classified point cloud. The histogram classification and region growing was programmed using Matlab 2011b, while the classification was performed using the LIBSVM, a SVM package developed by [35]. It was chosen due to its free access, easy use in Matlab and its availability on different platforms. We used Geomagic Studio only for preprocessing, labeling ground truth data and visualization purpose. The calculation was done on a computer including an Intel Core i7 processor (950 3.06 GHz) using 8 Gigabyte of RAM and Windows 7 64 Bit.

### Plant material

Grapevine plants (*Vitis vinifera* ssp. *vinifera*, variety Mueller Thurgau) and wheat plants (*Triticum aestivum*, variety Taifun) were grown in commercial substrate in plastic pots (∅ grapevine: 170 *mm*, wheat: 200 *mm*) under greenhouse conditions. Environmental parameters were kept constant at 23/20°C (day/night), 60% relative humidity and a photoperiod of 16 h. The plants were watered and fertilized on demand. Grapevine plants were used for laserscanning measurements at growth stage 19 (Laurenz et al. 1994), wheat plants were measured at the grain filling stage (Growth stage 85). The ears were harvested and the yield parameters ear weight, thousand kernel weight and the number of kernels were assessed. The chosen parameters are closely related to each other; the ear weight is influenced by the kernel weight and the number of kernels.

## Abbreviations

SVM: Support vector machines; CRF: Conditional random fields.

## Competing interests

The authors declare that they have no competing interests.

## Authors’ contributions

SP, AKM and HK designed the study. SP, AKM and JD interpreted the data and drafted the manuscript. SP and JD carried out the measuring and the programming. AKM maintained the plant material and the manual parameter extraction. HK directed the research and gave initial input. All authors read and approved the final manuscript.

## Supplementary Material

Additional file 1 **Wheat classification.** Avi video file using standart FFmpeg lossless video codec. Click here for file
